# AI, authorship teams and agency: Enacting fission and fusion

**DOI:** 10.1111/medu.70143

**Published:** 2025-12-13

**Authors:** Lauren A. Maggio, Abigail Konopasky

**Affiliations:** ^1^ Department of Medical Education University of Illinois, College of Medicine Chicago Illinois USA; ^2^ Department of Psychiatry Northwell Health New York New York USA

## Abstract

Always a delicate relationship, co‐authors has more ways to go wrong as AI becomes more prominent. This commentary asks if we are having the conversations we need to be having in our authorship teams.

In a recent study, Kıyak and colleagues found that explicitly warning medical students that ‘ChatGPT can make mistakes’ did not meaningfully change how they used it when making diagnostic decisions. Students in *both* the warned and unwarned groups showed similar scepticism and tended to underweight the tool's recommendations,^1^ suggesting that warnings alone do not meaningfully change behaviour.

We see a parallel in *research* practice, although, unfortunately, with less scepticism about AI. While authors recognise the warnings about artificial intelligence (AI) (e.g., it can hallucinate,^2^ introduce bias^3^), these warnings have not translated into changed behaviour, at least not for author team conversations. We know the risks but are not discussing them, considering their implications for the broader team or addressing how they shape our decisions about how and when we include AI. Unlike Kıyak's study, where diagnostic decisions were made individually, scholarly writing is inherently collective, so silence about AI use can create ethical and practical vulnerabilities for the whole team.

Increasingly, scholars are using AI,^4^ and journals are requiring disclosure, but few authors are actually disclosing its use.^5^ This underreporting may persist for various reasons. First, we lack shared habits and language to navigate these conversations and, thus, do not have them. Second, there is often shame and unease associated with admitting AI use, linked to fears of a ‘disclosure penalty’^6^: reviewers and editors questioning the work's quality or credibility.^7^

We have personally seen how AI can slip into research practice without collective agreement. Recently, one of us supervised a junior scholar who, right before submitting a manuscript, casually mentioned using AI to generate and polish the discussion. Some team members were surprised, others uneasy. What had seemed like a straightforward manuscript became an awkward ethical dilemma complicated by competing responsibilities to support a student, protect the team's reputation, align with journal guidelines and avoid jeopardising publication. This raised questions like: What about the integrity of the argument now known to be AI‐generated? Where did the student's intellectual contribution end and ChatGPT's begin? Because the team never discussed AI use, what should have been a shared decision became an individual one with implications for the entire team.

*What should have been a shared decision became an individual one with implications for the entire team*.

This conundrum can be framed as a problem of agency, in particular the negotiations around goals and behaviour that belong to the authorial ‘I’, the author team ‘we’ or various ‘they's’ like journals and readers. In 2022, we examined the ways this *distributed* agency is adopted in medical education publishing, showing how different teams navigated individual, joint and others' agency in authorship decisions.^8^ Kıyak's article prompted us to revisit this study, considering the role of AI. Through this lens, ‘they’ conversations are happening among journals, societies and institutions issuing policies and setting boundaries. Meanwhile, ‘I’ conversations are happening internally, as individuals privately use AI tools for scholarship. Finally, ‘we’ team conversations about how AI will be used are missing; this silence breeds misunderstanding and risks damaging author integrity.

Yet, our 2022 lens considers only one side of agency: *flexibility* (control over and design of action). Enfield and Kockelman^9^ argue that agency also includes *accountability*: being *evaluated* by others while also being *entitled* and/or *obligated* to certain actions. Kıyak's article highlights this critical gap in our understanding of agency: In our ‘we’ silence, we are not considering to whom we are *accountable* as authors: our co‐authors, our participants, the readers who might use our findings and the scientific record. Agency without accountability can lead to misunderstandings, risking the integrity and value of the work.

*Agency without accountability can lead to misunderstandings, risking the integrity and value of the work*.

Moving forward, scholars must shift their thinking about when and how their teams discuss AI use. Currently, AI conversations likely will arise only when the journal disclosure form appears during submission. By that point, it is too late. Teams should consider AI when starting a project, when establishing the team's ‘fission‐fusion dynamic[s]’,^9,p11^ breaking off for independent work and then coming together to work jointly with each other or tools like AI. Discussing these dynamics up front can clarify expectations for accountability, build trust and prevent awkward after‐the‐fact conversations. To facilitate this, we offer a few suggestions.

*Scholars must shift their thinking about when and how their teams discuss AI use*.

First, senior authors can model transparency, simply asking: ‘If we plan to use AI, how and for what purposes? To whom and what are we accountable as authors? How will we ensure integrity? How will we document and disclose that use?’ Asking these questions early allows everyone, including junior colleagues, to openly share their comfort levels and concerns. Research indicates that authorship decisions can put authors in vulnerable positions due to hierarchy, resource dependence, gender and institutional culture.^10^ And, given the differing comfort levels for scholarly AI use across junior and senior scholars, assumptions about ‘we’ actions with AI likely vary widely.^4^ Transparent, early dialogue kicked off by senior authors can help normalise these differences.

Second, journals could reframe AI‐disclosure guidelines. Rather than asking *whether* AI was used, editors might ask authors *how* and *when* teams discussed and decided upon AI use. Even a simple reflective prompt could significantly shift disclosure from a compliance task to an opportunity for collective reflection, reinforcing that accountability for AI use is a fused ‘we’ practice rather than a fissioned ‘I’ practice.

*Editors might ask authors* how *and* when *teams discussed and decided upon AI use*.

Third, we need training to practice AI conversations. At the 2025 Rogano Meeting, LM led a role‐play workshop for PhD students and supervisors to tackle exactly this (see Zenodo^11^ for materials). Participants stepped into the roles of learner, supervisor, middle author and journal editor to work through the dilemma presented above. Afterwards, participants shared that they had not previously considered how their team was using AI or how to communicate it proactively. Realising the increased prevalence of AI in scholarship, they valued experiencing multiple perspectives and exploring tensions among support, oversight and accountability. Moreover, both junior *and* senior scholars felt reluctance or shame about sharing AI use, not knowing how their colleagues might respond. Role play, case discussions and reflective exercises can normalise these conversations, preparing scholars to successfully navigate them.

*We need training to practice AI conversations*.

AI will continue to evolve faster than we can craft policies. Teams that can openly discuss AI use will be better equipped to respond to emerging norms and safeguard the integrity of their scholarship. Without conversations, we risk misunderstanding and potential harm. Kıyak and colleagues showed that warnings alone do not change behaviour. In their study, learners likely ignored the warnings because they were already cautious of AI. In author teams, silence around AI use may persist for different reasons (e.g., lack of shared norms, shame, fear of judgement), but with a similar outcome that warnings do not prompt change. Instead, what changes behaviour is reflection and conversation that considers not only where AI fits in the everyday fissions and fusions of team science but also to whom they are *accountable* as authorial agents.

*Coda*: As a team, we discussed whether and how to use AI. We decided to ask ChatGPT 5.0 to anticipate the impact of this commentary on readers: would it convince them of the problem? It massaged our egos telling us we ‘convincingly’ made our case, offering several suggestions. Based on our discussion of these, we added one clarifying sentence and reworded another. We take full responsibility for these changes.

## AUTHOR CONTRIBUTIONS

Lauren A. Maggio contributed to the conceptualization, writing of the original draft and the reviewing and editing of all subsequent drafts. Abigail Konopasky contributed to the conceptualization, writing of the original draft and the reviewing and editing of all subsequent drafts.

## CONFLICT OF INTEREST STATEMENT

None of the authors have a conflict of interest to disclose.

## ETHICS STATEMENT

Not applicable.

## Data Availability

None reported.
